# The Nature and Treatment of Acne*Read before Atlanta Medical Society, May, 1895.

**Published:** 1895-08

**Authors:** Bernard Wolff

**Affiliations:** Lecturer on Dermatology, Southern Medical College, Atlanta, Ga.


### THE NATURE AND TREATMENT OF ACNE.*

By BERNARD WOLFF, M. D.,

Lecturer on Dermatology, Southern Medical College, Atlanta, Ga.

The precise nature of acne has been for some years the subject of occasional discussion. Prior to the rise of bacteriology, as a constant means of investigating the cause and source of disease, the affection was exclusively regarded as a cutaneous manifestation of some systemic vice. But little thought was bestowed upon it as a local disorder, inherent in the skin itself. Since then, although many observations have been made of the micro-organisms that are constantly found and cultivated from the contents of the sebaceous gland and its excretory duct, in a pathological state, we are very little nearer an exact knowledge than before. No observer has yet had sufficient proof to warrant the confident assertion that any special form of micro-organism, however suggestive the constancy or uniformity of its occurrence may be, is the absolute and fixed cause of acne, and that it is alone sufficient to produce in a previously healthy skin indubitable evidences of primary occurrence of the disease. Moreover, it is certain that the clinical evidence deduced from observation is strong enough to forever set aside the possibility of such an explanation. But how gladly such an explanation would be received, and how it would clear away the uncertainties of diathesis, structural idiosyncracy and local predisposition.

The chemical evidence above referred to may be briefly considered, first, as regards the individual as a whole; and second, the skin as a part of the individual.

1. The masterly lectures of Jonathan Hutchinson, on the "Pedigree of Disease," delivered before the Royal College of Surgeons, some fifteen years ago, are marvels of astute reasoning, and clearly define the relation of temperament, diathesis, and idiosyncrasy to various diseased conditions. The least definite of the trio is temperament, which is to be considered as a physiological state, not implying a deviation from the normal, or a proclivity to disease, as is understood in the words diathesis and idiosyncrasy.

*Read before Atlanta Medical Society, May, 1895.

Of these two, the former may be briefly described as a proneness on the part of an individual to a particular form of disease, and the latter as a congenital peculiarity of an individual which does not indicate a tendency towards disease, but in which disease acts in an unusual and peculiar manner. We have, therefore, for the sake of example, the tubercular diathesis, in which there is a marked feebleness of resistance on the part of the organism against the invasion of the tubercle bacilli, and ichthyosis as a structural idiosyncrasy, and tobacco, morphine, and many other examples of the tendency toward abnormal manifestation in a definite and unfailing direction; the effect of these drugs upon the individual is unusual and fundamentally different from the effect upon the mass. Idiosyncrasy is, to borrow Hutchinson's definition, "diathesis brought to a point."

Now, let us briefly consider acne clinically, and then observe how the principle of a diathesis may be applied. The disease consists in an inflammatory condition of the sebaceous glands themselves and their excretory ducts, or in addition, an inflammation around the glands. As a result of inflammatory changes, there is a hypersecretion of their physiological product, and a break in the process by which this material is converted into a fluid, and is poured out on the skin or along the shaft of a hair. Though, to suit the requirements of hypersecretion, the excretory duct may be much dilated, it is unequal to the task and the thick secretion becomes blocked in the form of what is known as a comedone, which from the irritation offered by its presence as a foreign body, favors the occurrence of the minute inflammatory event in which the irritated point is converted into a pustule. The pustule finally ruptures, and the contents, including the plug of sebum, is discharged. The process of involution is complete, and the sebaceous gland regains its activity, after the subsidence of inflammation, or it is obliterated, and the seat of the pathological process is occupied by a small depressed scar, or a more or less persistent erythematous spot. While this constitutes the brief course of a single acne lesion, the process is repeated in the neighborhood, and the various stages of evolution and involution are continually going on. This occurs in those regions where the sebaceous glands are the most numerous, the face and back. The disease begins about the age of puberty, at the time when the development of the sexual system profoundly influences the whole economy. Through life the close intimacy between acne and the condition of the reproductive apparatus can be observed. This fact has been known for a long time, and gave rise to the expression, "*matrimonium varos curat.*" It was supposed, with some reason, that the regular sexual hygiene of married life would bring the organs of generation into their normal functional activity. But puberty, with its revolutionizing effect upon the organism, is not in itself, nor is any amount of reflex genital irritation, whether in the male or female, capable *per se* of determining the appearance of acne. The majority of mankind pass through this epoch without any or very slight and transient evidences of the disease, while others who had prior to this time been free from it, develop it in a marked and permanent form. Reflex genital irritation affects some individuals, so far as the skin is concerned, not at all; others, very much.

2. A necessary preliminary to the occurrence of acne concerns the second subdivision, structural peculiarity of the skin, inherited or congenital, not acquired. This peculiarity consists in a skin of lax fiber of more than ordinary thickness and coarseness, an exaggerated development and functional activity of the sebaceous glands, and a proclivity to glandular inflammation. This integumentary and glandular peculiarity occurs with striking frequency among those of the strumous habit, or the tubercular diathesis, in which a low resistance toward inflammation-arousing bacteria, is a prominent characteristic.

The special hue of the complexion has but little influence upon the occurrence of the disease, though in my experience, the cases among blondes have been much more frequent than among brunettes. Of thirty cases of acne, the majority of which were young girls treated by me in the last eighteen months, twenty-three have been of the blonde or rufous type.

Occupation has some influence; workers in tar are liable to it, and also those who are employed in candy factories or confectionery stores. Among eight young women emplo yees in a Whitehall street candy store, there are four who present more or less well-marked cases of acne; and at another similar establishment there are two out of five employees with acne, the remaining three suffering habitually from furuncles. This may be a coincidence, but if so, it is striking and suggestive. It may be due to fermentation in particles of powdered sugar which find entrance into the enlarged follicular openings in the faces of those predisposed to acne, and the effect is somewhat the same in contributing to the maintenance of the disease, as sugar taken internally.

Disorders of the digestive organs have a reflex influence upon the disease, but not in originating it. Indiscretion in diet, overloading the stomach, drinking malt liquors, sweet wine, the immoderate ingestion of pastries and candy, may in each instance give rise to fresh eruptions. In short, any reflex disturbance that serves to lower the tone of the system and that has a depressing effect upon the vitality, may, when occurring in an individual possessing a marked proclivity to the disease, cause an aggravation of the symptoms or light up an indolent and sluggish case of acne.

It is by no means difficult to reconcile the assumption of the diaethetic failure of the disease with the principle of its local origin. No one can be said to be naturally acneic, any more than that he is naturally rheumatic, malarious or syphilitic. Some potent factor or some peculiar circumstance is required for the development of the disease. It consists usually of an entrance into the skin through its several channels, of a living contagium of the active specific principle of a disease of special characterization, in which such an entrance is always followed by the same or very similar pathological results. Some plants require for their growth a clayey soil, others a sandy; and so vegetable micro-organisms differ only in degree from vegetable macro-organisms. A soil suitable for their growth and development is essential, without which they either do not take root at all, or their growth is abortive and their life insecure. If the sebaceous glands be in a state of normal functional activity, the skin sound, the vital elements of the body capable of resisting evil influences from without by reason of strength and perfect co-operation of individual parts, the danger of the invasion of micro-organisms would be reduced to a minimum or would cease to be a danger at all. But if the converse obtained, and the system were congenitally and habitually in a state of lowered vitality, or only the skin itself, entrance of a micro-organism such as the staphylococcus, and subsequent evidence of its activity could be easily understood, and acne could be placed, as has already been done, along with the local inoculable diseases, as impetigo contagiosa, sycosis, boils, carbuncle, malignant pustule and Delhi boils. We might say, finally, that acne is an inflammation of or around the sebaceous glands and their excretory ducts which arises from three conjoint sources, a diathetic predisposition on the part of the individual, a structural idiosyncrasy on the part of the skin, and a bacteriological source which is the active cause. Unless all the sources are present and the threefold condition fulfilled, the disease cannot exist.

Acne ranks very close to seborrhea. It is doubtful if there is ever a case of acne that does not show some seborrhœic features—more or less marked, and varying from a veritable seborrhœic eczema of the face to an unnatural oiliness, accompanied with erythema. If this condition cannot be found on the face, it will almost certainly be on the scalp. I believe it is now pretty generally admitted that seborrhea is a catarrh of the sebaceous or sweat glands, and not a mere hypersecretion of sebum, as it was formerly considered. If seborrhea be of inflammatory origin, its relation would be all the more near to acne and it would not be far wrong to say that acne is a concentrated type of seborrhea, plus a pyogenic micro-organism, probably the staphylococcus albus et aureus.

*Prognosis.* I do not believe that acne, except in mild cases, can be cured outright; that is, in the sense of *restitutio ad integrum*, but it can be more or less readily controlled and be kept under control. Up to late adult life, those who suffered in their youth from severe and especially untreated acne, must expect an occasional proruption of pustules. The general and local cutaneous peculiarities of such a person, invite the incursion of micro-organisms and until glandular atrophy that comes with old age of the skin has removed their nutrient soil, they will almost certainly show occasional activity. Even after the disease has disappeared, it is very apt to leave its mark in the shape of disfiguring pitting or a red and hypertrophied nose.

*Treatment.* The treatment is internal and local. The internal treatment is absolutely symptomatic—there is no rule for it. The various depressing influences from disorders of the digestive tract, the chylopöetic, and genito-urinary system must be counteracted by appropriate treatment. The relief of constipation and correction of diet are matters of importance. Cascara sagrada and nux vomica make an excellent tonic laxative. Bulkly's pill of sulphate of iron and aloes is of service as a laxative. Ichthyol in two-drop doses in a capsule is an excellent remedy for fermentative dyspepsia. The diet should be plain, avoiding such articles as are apt to cause flatulence and fermentative dyspepsia, as this condition causes a reflex congestion of the face and is deleterious. I have seen good results accrue from the use of the solution of bromide of gold and arsenic known as arsenauro, particularly in relieving the annoying flushing of the face in acne with rosacea. The local treatment is to be mainly relied upon. I suppose as there are so many methods of treating acne that each one makes his own selection according to individual preference or experience. My practice has been at the very beginning of treatment, to curette the skin of the face thoroughly, in order to tear off the tops of the pustules and liberate the contents as well as drag out the comedones from the sebaceous gland ducts. The larger pustules should be incised with an acne lancet and the contents expressed and the larger comedones pushed out with a comedo extractor or between the thumb nails.

After having treated the face in this way, I order a sulphur lotion, which is very well known and is certainly one of the best. It consists of:

℞ Zinc Sulphate, Potassium Sulphuret, aa ʒj. Aq. Rosae, ℥iij, Sulph. Praecip, ʒj.

The amount of the ingredients can be increased or diminished as desired. I think the lotion owes its value to the precipitated sulphur contained. This is to be applied every night after having first washed the face with tepid water to which borax has been added.

If the sulphur be too drying and the face becomes scaly, an ointment of Boric acid gr. x., Pulv. Camph. gr. v, to cold cream ℥j. is used until the skin has regained its pliancy.

If this does not remove the eruption in several weeks, the following salve is recommended to be used at night, and during the day, the face powdered with the compound stearate of zinc:

| | | |
|---|---|---|
| Hydrarg Bichlor gr.......... | 1—ij. | |
| Resorcin..................... | ʒss–j. | |
| Aq. Lauro-Cerasi............. | ʒij. | |
| Farinæ....................... | ʒij. | |
| Laneolin ad.................. | ℥j. | M. Ft. ung. |

This should be used for some time. If the lesions are sluggish and indolent and approaching the indurated form, an ointment of resorcin, ichthyol and sulphur aa ʒj. to the ounce of lanolin is prescribed; this will often abort fresh or beginning eruptions.

In acne indurata, the deep-seated lesions should be incised and the contents squeezed out as far as possible, and the last named ointment used. Betanaphthol in the form of an ointment 5 to 10 % is of service in like cases and also Unna's mercury-carbolic plaster-mull, a specimen of which I here present.

Curetting the face thoroughly every week or ten days is of great benefit. The disfigurement caused by it is very transient, as the horny layer is readily and rapidly replaced.

Electricity, in the chronic, indurated form, is of some use. The electrodes of a constant current battery are applied to the two sides of the face and a current of 5-8 milliamperes used for ten or fifteen minutes. The electrodes had best be moistened in a 1-1000 solution of bichloride and their position frequently shifted so as not to cause too much hyperæmia.

All remedies used in the local treatment of acne are antiseptics of different degrees of strength, and the choice is determined by the amount of antiseptic influence desired.

Steaming the face and the use of very hot water do harm as they increase seborrhea and greatly favor the growth of germs.

Soap and water for the sake of cleanliness should be freely used throughout the whole course of treatment. An ordinary transparent toilet soap is doubtless as good as any of the so-called medicated soaps, with the exception of carbolic soap. In my experience carbolic soap alone is sufficient in the treatment of acne of the back. Frequent baths should be taken and the lather of the soap allowed to dry on the back. Rarely anything more than cleanliness and some mild antiseptic is necessary to relieve acne of the back, in which situation it is more than likely to be due to neglect of personal cleanliness.

Comedones are quite often difficult to remove permanently. Mechanical extraction of them is the best method, and to guard against recurrences, Hebra's Spiritus Saponatus Kalinus, or the less elegant tincture of green soap should be rubbed in on a bit of cotton, with considerable friction. This can be repeated twice a week but not pushed to the point of causing an exfoliation of the epidermis.

